# Supplementary material for: Mutations that improve efficiency of a weak-link enzyme are rare compared to adaptive mutations elsewhere in the genome
Source: eLife. 2019 Dec 9;8:e53535. doi: 10.7554/eLife.53535 (PMC6941894; doi:10.7554/eLife.53535)
Supplement: Supplementary file 1. [file elife-53535-supp1.docx]

# Supplementary file 1: Plasmids used in this study

| Plasmid | Use / description | Source/Ref |
| --- | --- | --- |
| pSLTS | scarless genome editing / arabinose-inducible 𝜆 Red recombinase, anhydrotetracycline-inducible meganuclease I-SceI, temperature-sensitive origin of replication, ampicillin resistance | (Kim et al., 2014) |
| pSIM5 | introduction of linear DNA fragments into the genome/heat-inducible 𝜆 Red recombinase / temperature-sensitive origin of replication, chloramphenicol resistance | (Datta et al., 2006) |
| pSIM27 | introduction of linear DNA fragments into the genome/heat-inducible 𝜆 Red recombinase / temperature-sensitive origin of replication, tetracycline resistance | (Datta et al., 2006) |
| SS9_RNA | Cas9 guide RNA (expressed under control of the J23119 promoter) targeting safe site 9 (SS9) in the *E. coli* genome (Bassalo et al., 2016), ampicillin resistance | Addgene #71656 (Bassalo et al., 2016) |
| pET-46 | backbone for IPTG-inducible expression of wild-type and mutant ProAs and ArgB / T7 promoter upstream of 6xHis-tag, ampicillin resistance | Novagen |
| pACYC177 | low-copy plasmid backbone, ampicillin and kanamycin resistance |  |
| pCA24N | backbone for IPTG-inducible expression of wild-type and mutant carbamoyl phosphate synthetases / chloramphenicol resistance | (Kitagawa et al., 2005) |
| pCA24N-*argI* | IPTG-inducible expression of ornithine transcarbamoylase / chloramphenicol resistance | (Kitagawa et al., 2005) |
| pCA24N-*argD* | IPTG-inducible expression of *N*-acetylornithine aminotransferase / chloramphenicol resistance | (Kitagawa et al., 2005) |
| pAM003 | pSB1A2 vector expressing CFP under control of the pTet promoter (Biobrick part BBa_I13600), ampicillin resistance | iGEM |
| pAM027 | IPTG-inducible expression of ArgC with N-terminal 6xHis-tag + Gly-Met-Ala-Ser linker with Met1 removed / pTrcHisB backbone, ampicillin resistance | (McLoughlin & Copley, 2008) |
| pAM028 | IPTG-inducible expression of ArgB with N-terminal 6xHis-tag + Val-Val linker / pET-46 backbone, ampicillin resistance | This study |
| pAM053 | Cas9-mediated genome editing / constitutively-expressed c*as9*, heat-inducible 𝜆 Red recombinase, temperature-sensitive origin of replication, chloramphenicol resistance | This study |
| pAM063 | IPTG-inducible expression of ProA with N-terminal 6xHis-tag + Val-Val linker / pET-46 backbone, ampicillin resistance | This study |
| pAM064 | IPTG-inducible expression of E383A ProA with N-terminal 6xHis-tag + Val-Val linker / pET-46 backbone, ampicillin resistance | This study |
| pAM068 | Cas9 guide RNA (J23119 promoter) that targets the region upstream of *argB* for introduction of a 58 bp deletion upstream of *argB* / temperature-sensitive origin of replication and ampicillin resistance from pSLTS, guide RNA and promoter from SS9_RNA plasmid, guide RNA protospacer from SS9_plasmid modified by site-directed mutagenesis to target the region upstream of *argB* (NEB, primers in Table S2, protospacer sequence in Table S3) | This study |
| pAM078 | IPTG-inducible expression of ArgC with N-terminal 10xHis-tag + SUMO-tag by T7 polymerase / pET28 backbone, kanamycin resistance | This study |
| pAM100 | Cas9 guide RNA (J23119 promoter) that targets *rph* for introduction of the 82 bp deletion upstream of *pyrE* / temperature-sensitive origin of replication and ampicillin resistance from pSLTS, guide RNA protospacer from pAM068 modified by site-directed mutagenesis to target *rph* (primers in Table S2, protospacer sequence in Table S3) | This study |
| pAM101 | IPTG-inducible expression of CarAB with N-terminal 6xHis-tag on CarA / pCA24N backbone, chloramphenicol resistance | This study |
| pAM102 | IPTG-inducible expression of CarA and G369V CarB with N-terminal 6xHis-tag on CarA / pCA24N backbone, chloramphenicol resistance | This study |
| pAM103 | IPTG-inducible expression of CarA and L960P CarB with N-terminal 6xHis-tag on CarA / pCA24N backbone, chloramphenicol resistance | This study |
| pAM104 | IPTG-inducible expression of CarA and L964Q CarB with N-terminal 6xHis-tag on CarA / pCA24N backbone, chloramphenicol resistance | This study |
| pAM105 | IPTG-inducible expression of CarA and K966E CarB with N-terminal 6xHis-tag on CarA / pCA24N backbone, chloramphenicol resistance | This study |
| pAM106 | IPTG-inducible expression of CarA and CarB (12 bp deletion at nt 2906) with N-terminal 6xHis-tag on CarA / pCA24N backbone, chloramphenicol resistance | This study |
| pAM107 | IPTG-inducible expression of CarA and CarB (132 bp deletion at nt 2986) with N-terminal 6xHis-tag on CarA / pCA24N backbone, chloramphenicol resistance | This study |
| pAM108 | IPTG-inducible expression of CarA and CarB (12 bp deletion at nt 3108) with N-terminal 6xHis-tag on CarA / pCA24N backbone, chloramphenicol resistance | This study |
| pAM109 | IPTG-inducible expression of CarA and CarB (21 bp duplication at nt 3145) with N-terminal 6xHis-tag on CarA / pCA24N backbone, chloramphenicol resistance | This study |
| pAM112 | IPTG-inducible expression of F372L E383A ProA with N-terminal 6xHis-tag + Val-Val linker / pET-46 backbone, ampicillin resistance | This study |
| pAM116 | expresses the Cas9 guide RNA (J23119 promoter) that targets *carB* for introduction of the 12 bp deletion at nt 2906 / temperature-sensitive origin of replication and ampicillin resistance from pSLTS, guide RNA protospacer from pAM068 modified by site-directed mutagenesis to target *carB* (primers in Table S2, protospacer sequence in Table S3) | This study |
| pAM117 | expresses the Cas9 guide RNA (J23119 promoter) that targets *carB* for introduction of the 132 bp deletion at nt 2986 / temperature-sensitive origin of replication and ampicillin resistance from pSLTS, guide RNA protospacer from pAM068 modified by site-directed mutagenesis to target *carB* (primers in Table S2, protospacer sequence in Table S3) | This study |
| pAM128 | Encodes the *argC(null)* allele with G153 and C154 changed to TAA stop codons / used for amplifying *argC(null)* mutation cassette, generated using site-directed mutagenesis of pAM078 (primers in Table S2) | This study |
| pAM129 | expresses the Cas9 guide RNA (J23119 promoter) that targets *kan^r^* for replacement with the *argC(null)* allele / temperature-sensitive origin of replication and ampicillin resistance from pSLTS, guide RNA protospacer from pAM068 modified by site-directed mutagenesis to target *kan^r^* (primers in Table S2, protospacer sequence in Table S3) | This study |
| pAM141 | *argB* expressed under control of the native *argCBH* promoter / pACYC177 backbone (low-copy), ampicillin resistance | This study |
| pAM142 | same as pAM003 but expressing eYFP instead of CFP | This study |
