# Supplementary material for: Mutations that improve efficiency of a weak-link enzyme are rare compared to adaptive mutations elsewhere in the genome
Source: eLife. 2019 Dec 9;8:e53535. doi: 10.7554/eLife.53535 (PMC6941894; doi:10.7554/eLife.53535)
Supplement: Supplementary file 2. [file elife-53535-supp2.docx]

# Supplementary file 2: Primers used in this study

| Name | sequence (5’⟶3’) | description |
| --- | --- | --- |
| 1357 | CGTGCAGGCGATTGATAA | Forward primer for *proA* qPCR |
| 1358 | CTGTTCACGGCACAGTTT | Reverse primer for *proA* qPCR |
| 1359 | CGTAGATCTGACGGTGAATTT | Forward primer for *gyrB* qPCR |
| 1360 | CGTTGGTGTTTCGGTAGTA | Reverse primer for *gyrB* qPCR |
| 1361 | CCCGTGGCTGAAAGTTAAA | Forward primer for *icd* qPCR |
| 1362 | CAGGTTCATACAGGCGATAAC | Reverse primer for *icd* qPCR |
| hcaT_F | CGTGGTGGCGGAAGTCATTATC | Forward primer for *hcaT* RT-qPCR |
| hcaT_R | CGCCGAGATCAACAGCATATCG | Reverse primer for *hcaT* RT-qPCR |
| cysG_F | GGTGGCGAAGAGCTGGAAA | Forward primer for *cysG* RT-qPCR |
| cysG_R | GAATACCCGAATAGGCAGAGCAA | Reverse primer for *cysG* RT-qPCR |
| argB_F | GGCGGGAACGGCAAATAAAA | Forward primer for *argB* RT-qPCR |
| argB_R | CCGTCACCGAGAAACAAACC | Reverse primer for *argB* RT-qPCR |
| argH_F | TGGTGGAAACCGCACAAAAC | Forward primer for *argH* RT-qPCR |
| argH-R | GCGCCAGCATCTCAACATAG | Reverse primer for *argH* RT-qPCR |
| rAM069 | ACCACCACCATCACGTGGATATGAATCCATTAATTATCAAACTGGGCG | Forward primer for amplification and cloning of *argB* to make plasmid pAM028 |
| rAM070 | CTTTACCAGACTCGAGATTTTTAAGCTAAAATCCGCGTACCCATCG | Reverse primer for amplification and cloning of *argB* to make plasmid pAM028 |
| rAM199 | ACCACCACCATCACGTGGTGATGCTGGAACAAATGGGCATTG | Forward primer for amplification and cloning of *proA* and *proA** to make plasmids pAM063 and pAM064, respectively |
| rAM200 | CTTTACCAGACTCGAGATTTTTACGCACGAATGGTGTAATCAC | Reverse primer for amplification and cloning of *proA* and *proA** to make plasmids pAM063 and pAM064, respectively |
| rAM340 | CTACGCGTTTGACCGACGGCG | Forward primer for introduction of the *proA*** mutation by site-directed mutagenesis (pAM112) |
| rAM341 | AGGCGTTAACGTAAACAGCG | Reverse primer for introduction of the *proA*** mutation by site-directed mutagenesis (pAM112) |
| rAM315 | CGGATCCGGCCCTGAGGGCCATTAAGTCAGCGCTATTGGT | Forward primer for amplification and cloning of *carAB* to make plasmid pAM101 |
| rAM316 | GCTTGGCTGCAGGTCGACCCTTATTTGATCTGTGCGTGCA | Reverse primer for amplification and cloning of *carAB* to make plasmid pAM101 |
| rAM317 | GGGTCGACCTGCAGCC | Forward primer for amplification of pCA24N backbone |
| rAM318 | GGCCCTCAGGGCCGGA | Reverse primer for amplification of pCA24N backbone |
| rAM322 | AAATTCGCCGTTGCTAACGAC | Forward primer for introduction of the G369V CarB mutation by site-directed mutagenesis of pAM101 to generate pAM102 |
| rAM323 | TTCGAAGTTGAAGCGAGG | Reverse primer for introduction of the G369V CarB mutation by site-directed mutagenesis of pAM101 to generate pAM102 |
| rAM324 | GTGGTGGACCCGGCGGCAAAA | Forward primer for introduction of the L960P CarB mutation by site-directed mutagenesis of pAM101 to generate pAM103 |
| rAM325 | GCGTTCTTTATCGCCTTCG | Reverse primer for introduction of the L960P CarB mutation by site-directed mutagenesis of pAM101 to generate pAM103 |
| rAM326 | GCGGCAAAACAGCTGAAACAGG | Forward primer for introduction of the L964Q CarB mutation by site-directed mutagenesis of pAM101 to generate pAM104 |
| rAM327 | CAGGTCCACCACGCGTTC | Reverse primer for introduction of the L964Q CarB mutation by site-directed mutagenesis of pAM101 to generate pAM104 |
| rAM328 | AAAACTGCTGGAACAGGGCTTCGAGC | Forward primer for introduction of the K966E CarB mutation by site-directed mutagenesis of pAM101 to generate pAM105 |
| rAM329 | GCCGCCAGGTCCACCACG | Reverse primer for introduction of the K966E CarB mutation by site-directed mutagenesis of pAM101 to generate pAM105 |
| rAM330 | CGACCCACGGCACGGCGA | Forward primer for introduction of the 12 bp deletion at nt 2906 *carB* mutation by site-directed mutagenesis of pAM101 to generate pAM106 |
| rAM331 | AGCCCTGTTTCAGCAGTTTTGCCG | Reverse primer for introduction of the 12 bp deletion at nt 2906 *carB* mutation by site-directed mutagenesis of pAM101 to generate pAM106 |
| rAM332 | TACGACACCACCCTGAAC | Forward primer for introduction of the 132 bp deletion at nt 2986 *carB* mutation by site-directed mutagenesis of pAM101 to generate pAM107 |
| rAM333 | ATGCACCTTGTTTACCAG | Reverse primer for introduction of the 132 bp deletion at nt 2986 *carB* mutation by site-directed mutagenesis of pAM101 to generate pAM107 |
| rAM334 | CGACACCACCCTGAACGG | Forward primer for introduction of the 12 bp deletion at nt 3108 *carB* mutation by site-directed mutagenesis of pAM101 to generate pAM108 |
| rAM335 | TATTGCAGCGCACTGCGA | Reverse primer for introduction of the 12 bp deletion at nt 3108 *carB* mutation by site-directed mutagenesis of pAM101 to generate pAM108 |
| rAM336 | GGCGGCTTTGCCACCGCGATGGCGCTGA | Forward primer for introduction of the 21 bp duplication at nt 3145 *carB* mutation by site-directed mutagenesis of pAM101 to generate pAM109 |
| rAM337 | GTTCAGGGTGGCAAAGCCGCCGTTCAGGG | Reverse primer for introduction of the 21 bp duplication at nt 3145 *carB* mutation by site-directed mutagenesis of pAM101 to generate pAM109 |
| rAM209 | GACATAGCGTTGGCTACCCG | Forward primer for amplifying linear mutation fragment for the 58 bp deletion upstream of *argB* |
| rAM192 | CAGCCCTTTCATCAGCTCATCC | Reverse primer for amplifying linear mutation fragment for the 58 bp deletion upstream of *argB* |
| rAM307 | AGCGGTTTGCGATCTGGAAT | Forward primer for amplifying linear mutation fragment for the 82 bp deletion in *rph* upstream of *pyrE* |
| rAM308 | ATGGTTTCATGCCTTCGCTC | Reverse primer for amplifying linear mutation fragment for the 82 bp deletion in *rph* upstream of *pyrE* |
| rAM369 | AACTCCACCATGAAGAAACA | Forward primer for amplifying linear mutation fragment for the 12 bp deletion in *carB* at nt 2906 |
| rAM370 | TTGATACGGTCCTGAATGTG | Reverse primer for amplifying linear mutation fragment for the 12 bp deletion in *carB* at nt 2906 |
| rAM371 | AAAACTGCTGAAACAGGGC | Forward primer for amplifying linear mutation fragment for the 132 bp deletion in *carB* at nt 2986 |
| rAM372 | TTATTTGATCTGTGCGTGCA | Reverse primer for amplifying linear mutation fragment for the 132 bp deletion in *carB* at nt 2986 |
| rAM382 | TTTAACCTGGCAACCAGACATAAGAAGGTGAATAGCCCCGATGTTGAATACGCTGATTGT | Forward primer for amplifying linear fragment for replacing *kan^r^* with the *argC(null)* allele |
| rAM383 | GCCGCCCAGTTTGATAATTAATGGATTCATCATTGCACCCTTAAATAAGAGACTGCGTTT | Reverse primer for amplifying linear fragment for replacing *kan^r^* with the *argC(null)* allele |
| rAM109 | TTTTTACGGTTCCTGGCCTT | Forward primer for amplifying Cas9 guide RNA and associated promoter from SS9_RNA |
| rAM110 | GATTATCAAAAAGGATCTTCACCTAG | Reverse primer for amplifying Cas9 guide RNA and associated promoter from SS9_RNA |
| rAM111 | AAGGCCAGGAACCGTAAAAATATGGACAGTTTTCCCTTTGAT | Forward primer for amplifying origin of replication and ampicillin resistance gene from pSLTS for Gibson assembly of pAM068 |
| rAM112 | CTAGGTGAAGATCCTTTTTGATAATCGTTGATGATACCGCTGCCTT | Reverse primer for amplifying origin of replication and ampicillin resistance from pSLTS for Gibson assembly of pAM068 |
| rAM210 | TTCTGTAGGCGTTTTAGAGCTAGAAATAGC | Forward primer for site-directed mutagenesis of guide RNA protospacer for pAM068 |
| rAM211 | ACGCAGTCTCACTAGTATTATACCTAGGAC | Reverse primer for site-directed mutagenesis of guide RNA protospacer for pAM068 |
| rAM313 | TTGTTGGCTCGTTTTAGAGCTAGAAATAGC | Forward primer for site-directed mutagenesis of guide RNA protospacer for pAM100 |
| rAM314 | GATGAGTAGCACTAGTATTATACCTAGGAC | Reverse primer for site-directed mutagenesis of guide RNA protospacer for pAM100 |
| carB_2906gRNA_F | GGCTTCGAGCGTTTTAGAGCTAGAAATAGC | Forward primer for site-directed mutagenesis of guide RNA protospacer for pAM116 |
| carB_2906gRNA_R | CTGTTTCAGCACTAGTATTATACCTAGGAC | Reverse primer for site-directed mutagenesis of guide RNA protospacer for pAM116 |
| carB_2986gRNA_F | AATGTGCGGAGTTTTAGAGCTAGAAATAGC | Forward primer for site-directed mutagenesis of guide RNA protospacer for pAM117 |
| carB_2986gRNA_R | CAGGACCGTAACTAGTATTATACCTAGGAC | Reverse primer for site-directed mutagenesis of guide RNA protospacer for pAM117 |
| rAM384 | GGCTATGACTGTTTTAGAGCTAGAAATAGC | Forward primer for site-directed mutagenesis of guide RNA protospacer for pAM129 |
| rAM385 | GAATAGCCTCACTAGTATTATACCTAGGAC | Reverse primer for site-directed mutagenesis of guide RNA protospacer for pAM129 |
| rAM380 | TAATAATATCCGACGGCGGCACAG | Forward primer for site-directed mutagenesis of *argC(null)* for pAM128 |
| rAM381 | CGGCACCGCAATCAAATTCG | Reverse primer for site-directed mutagenesis of *argC(null)* for pAM128 |
