# Supplementary material for: Mutations that improve efficiency of a weak-link enzyme are rare compared to adaptive mutations elsewhere in the genome
Source: eLife. 2019 Dec 9;8:e53535. doi: 10.7554/eLife.53535 (PMC6941894; doi:10.7554/eLife.53535)
Supplement: Supplementary file 3. [file elife-53535-supp3.docx]

# Supplementary file 3: Protospacers used for Cas9-mediated genome editing.

| Genome modification | plasmid | protospacer (5’⟶3’) |
| --- | --- | --- |
| 58 bp deletion upstream of *argB* | pAM068 | GAGACTGCGTTTCTGTAGGC |
| 82 bp deletion in *rph* upstream of *pyrE* | pAM100 | GCTACTCATCTTGTTGGCTC |
| 12 bp deletion in *carB* at nt 2906 | pAM116 | GCTGAAACAGGGCTTCGAGC |
| 132 bp deletion in *carB* at nt 2986 | pAM117 | TACGGTCCTGAATGTGCGGA |
| *kan^r^*::*argC(null)* | pAM129 | GAGGCTATTCGGCTATGACT |
