# Supplementary material for: Mutations that improve efficiency of a weak-link enzyme are rare compared to adaptive mutations elsewhere in the genome
Source: eLife. 2019 Dec 9;8:e53535. doi: 10.7554/eLife.53535 (PMC6941894; doi:10.7554/eLife.53535)
Supplement: Supplementary file 4. [file elife-53535-supp4.docx]

# Supplementary file 4: Mutation cassettes used for Cas9-mediated genome editing. Brackets represent the primer annealing regions for amplifying the mutation cassettes from either genomic DNA of clones isolated during the evolution experiment or from plasmids that contained sequences with the desired deletions.

| Genome modification | sequence (5’⟶3’) | PCR template | |
| --- | --- | --- | --- |
| 58 bp deletion upstream of *argB* | [GACATAGCGTTGGCTACCCG]TGATATTGCTGAAGAGCTTGGCGGCGAATGGGCTGACCGCTTCCTCGTGCTTTACGGTATCGCCGCTCCCGATTCGCAGCGCATCGCCTTCTATCGCCTTCTTGACGAGTTCTTCTAATAAGGGGATCTTGAAGTTCCTATTCCGAAGTTCCTATTTTAAGGGTGCAATGATGAATCCATTAATTATCAAACTGGGCGGCGTACTGCTGGATAGTGAAGAGGCGCTGGAACGTCTGTTTAGCGCACTGGTGAATTATCGTGAGTCACATCAGCGTCCGCTGGTGATTGTGCACGGCGGCGGTTGCGTGGT[GGATGAGCTGATGAAAGGGCTG] | population 6 genomic DNA |  |
| 82 bp deletion in *rph* upstream of *pyrE* | [AGCGGTTTGCGATCTGGAAT]ACGTTGAAGACTCTGCCGCAGAGACCGACATGAACGTAGTGATGACCGAAGACGGGCGCATCATTGAAGTGCAGGGGACGGCAGAAGGCGGCGCTGGCAAACTGATTTTTAAGGCGACTGATGAGTCGCCTTTTTTTTGTCTGTAGAAAAGTAAGATGAG[GAGCGAAGGCATGAAACCAT] | population 1 genomic DNA |  |
| 12 bp deletion in *carB* at nt 2906 | [AACTCCACCATGAAGAAACA]CGGTCGTGCGCTGCTTTCCGTGCGCGAAGGCGATAAAGAACGCGTGGTGGACCTGGCGGCAAAACTGCTGAAACAGGGCTCGACCCACGGCACGGCGATTGTGCTGGGCGAAGCAGGTATCAACCCGCGTCTGGTAAACAAGGTGCATGAAGGCCGTCCG[CACATTCAGGACCGTATCAA] | pAM106 |  |
| 132 bp deletion in *carB* at nt 2986 | [AAAACTGCTGAAACAGGGC]TTCGAGCTGGATGCGACCCACGGCACGGCGATTGTGCTGGGCGAAGCAGGTATCAACCCGCGTCTGGTAAACAAGGTGCATTACGACACCACCCTGAACGGCGGCTTTGCCACCGCGATGGCGCTGAATGCCGATGCGACTGAAAAAGTAATTTCGGTGCAGGAAA[TGCACGCACAGATCAAATAA] | pAM107 |  |
| *kan^r^*::*argC(null)* | [TTTAACCTGGCAACCAGACATAAGAAGGTGAATAGCCCCGATGTTGAATACGCTGATTGT]GGGTGCCAGCGGCTACGCTGGCGCAGAGCTAGTGACCTATGTAAATCGCCATCCGCATATGAACATAACCGCTTTGACTGTTTCAGCGCAAAGCAATGATGCGGGAAAGTTAATCTCCGATTTGCATCCGCAGCTAAAAGGCATCGTTGATCTGCCGTTGCAGCCGATGTCGGATATCAGCGAGTTTAGCCCAGGGGTGGACGTAGTGTTTCTCGCCACCGCCCATGAAGTTAGCCACGATTTAGCGCCGCAGTTTCTTGAAGCGGGCTGCGTGGTGTTCGACCTTTCCGGCGCGTTTCGTGTTAACGACGCCACCTTCTATGAAAAATATTACGGCTTTACCCATCAATACCCGGAACTGTTGGAACAGGCAGCCTACGGTCTGGCGGAGTGGTGCGGTAATAAATTAAAAGAAGCGAATTTGATTGCGGTGCCGtaataaTATCCGACGGCGGCACAGCTGGCGCTGAAACCGTTGATTGATGCCGATCTTCTTGACCTCAATCAGTGGCCGGTGATCAACGCCACCAGCGGCGTGAGCGGTGCAGGGCGTAAAGCGGCCATTTCAAACAGCTTTTGTGAAGTTAGCCTGCAACCGTATGGCGTCTTTACTCATCGCCATCAACCAGAGATCGCCACACACCTCGGTGCTGACGTTATCTTCACCCCACATCTGGGCAATTTCCCGCGCGGCATTCTCGAAACCATTACCTGCCGCCTGAAATCGGGTGTGACCCAGGCGCAAGTCGCGCAAGTGTTACAGCAGGCGTATGCCCATAAACCGCTGGTGCGGCTGTATGACAAAGGCGTTCCGGCGCTGAAAAATGTCGTTGGGCTGCCATTTTGCGATATCGGGTTTGCCGTTCAGGGCGAGCATCTGATTATTGTGGCGACCGAAGACAACTTACTGAAAGGCGCGGCGGCACAAGCGGTACAGTGCGCCAATATTCGTTTCGGCTATGCGG[AAACGCAGTCTCTTATTTAAGGGTGCAATGATGAATCCATTAATTATCAAACTGGGCGGC] | pAM128 |  |
