# Supplementary material for: Mutations that improve efficiency of a weak-link enzyme are rare compared to adaptive mutations elsewhere in the genome
Source: eLife. 2019 Dec 9;8:e53535. doi: 10.7554/eLife.53535 (PMC6941894; doi:10.7554/eLife.53535)
Supplement: Transparent reporting form [file elife-53535-transrepform.docx]

***eLife’s* transparent reporting form**

We encourage authors to provide detailed information *within their submission* to facilitate the interpretation and replication of experiments. Authors can upload supporting documentation to indicate the use of appropriate reporting guidelines for health-related research (see [EQUATOR Network](http://www.equator-network.org/%20)), life science research (see the [BioSharing Information Resource](https://biosharing.org/)), or the [ARRIVE guidelines](http://www.plosbiology.org/article/info:doi/10.1371/journal.pbio.1000412) for reporting work involving animal research. Where applicable, authors should refer to any relevant reporting standards documents in this form.

If you have any questions, please consult our Journal Policies and/or contact us: [editorial@elifesciences.org](mailto:editorial@elifesciences.org).

**Sample-size estimation**

- You should state whether an appropriate sample size was computed when the study was being designed
- You should state the statistical method of sample size computation and any required assumptions
- If no explicit power analysis was used, you should describe how you decided what sample (replicate) size (number) to use

Please outline where this information can be found within the submission (e.g., sections or figure legends), or explain why this information doesn’t apply to your submission:

Because we performed evolution experiments using *E. coli*, this sort of power analysis was not necessary. Evolution has an element of contingency due to the random acquisition of mutations. We performed 8 parallel adaptations from the same starting clone; each population is inherently unique in its evolutionary trajectory. Replicates in adaptive evolution experiments such as ours are critical for identifying mutations that appear in multiple lineages, which are most likely to be adaptive rather than neutral hitchhikers. The fitness effects of the most common potentially adaptive mutations were tested directly as described in the text.

**Replicates**

- You should report how often each experiment was performed
- You should include a definition of biological versus technical replication
- The data obtained should be provided and sufficient information should be provided to indicate the number of independent biological and/or technical replicates
- If you encountered any outliers, you should describe how these were handled
- Criteria for exclusion/inclusion of data should be clearly stated
- High-throughput sequence data should be uploaded before submission, with a private link for reviewers provided (these are available from both GEO and ArrayExpress)

Please outline where this information can be found within the submission (e.g., sections or figure legends), or explain why this information doesn’t apply to your submission:

Biological replicate: protocol repeated from start to finish, beginning with the growth of a bacterial clone from a frozen glycerol stock. All sample sizes (N) reported in the figure legends refer to biological replicates. Standard errors or standard deviations reported for RT-qPCR, growth rate, and proteomics were calculated from the variance between biological replicates.

Technical replicate: quantification of a sample repeated on the same instrument on the same day. Technical replicates of a biological replicate were averaged to produce the final value for a biological replicate. In the case of enzyme kinetics, the values reported are the averages/standard errors of technical replicates using the same purified enzyme. Information on the number of technical replicates used for enzyme kinetics experiments can be found in the table and figure legends.

Outliers: technical replicates were used to account for any outliers that may have been caused by instrument variation or poor manual preparation. Technical replicate outliers were excluded from the final average calculation only if there was sufficient evidence for an instrument or preparation error.

**Statistical reporting**

- Statistical analysis methods should be described and justified
- Raw data should be presented in figures whenever informative to do so (typically when N per group is less than 10)
- For each experiment, you should identify the statistical tests used, exact values of N, definitions of center, methods of multiple test correction, and dispersion and precision measures (e.g., mean, median, SD, SEM, confidence intervals; and, for the major substantive results, a measure of effect size (e.g., Pearson's r, Cohen's d)
- Report exact p-values wherever possible alongside the summary statistics and 95% confidence intervals. These should be reported for all key questions and not only when the p-value is less than 0.05.

Please outline where this information can be found within the submission (e.g., sections or figure legends), or explain why this information doesn’t apply to your submission:

Any time a test for statistical significance was performed, the exact statistical method used is described in the figure legend.

Standard error was calculated by σ/√(*n*) where σ is the standard deviation and *n* is the sample size.

(For large datasets, or papers with a very large number of statistical tests, you may upload a single table file with tests, Ns, etc., with reference to sections in the manuscript.)

**Group allocation**

- Indicate how samples were allocated into experimental groups (in the case of clinical studies, please specify allocation to treatment method); if randomization was used, please also state if restricted randomization was applied
- Indicate if masking was used during group allocation, data collection and/or data analysis

Please outline where this information can be found within the submission (e.g., sections or figure legends), or explain why this information doesn’t apply to your submission:

Group allocation is not relevant to this type of experimental evolution study.

**Additional data files (“source data”)**

- We encourage you to upload relevant additional data files, such as numerical data that are represented as a graph in a figure, or as a summary table
- Where provided, these should be in the most useful format, and they can be uploaded as “Source data” files linked to a main figure or table
- Include model definition files including the full list of parameters used
- Include code used for data analysis (e.g., R, MatLab)
- Avoid stating that data files are “available upon request”

Please indicate the figures or tables for which source data files have been provided:

We have provided the R script used to generate the data in Figure 3. This script was used to calculate growth rate for each population in real time from the turbidostat OD readings.

We have provided source data for Figure 4 showing all the mutations identified in the adapted populations.

We have provided example R scripts used to calculate the enzyme kinetics constants shown in Tables 2 and 3.
